# Transportation Safety of Lithium Iron Phosphate Batteries - A Feasibility Study of Storing at Very Low States of Charge

**DOI:** 10.1038/s41598-017-05438-2

**Published:** 2017-07-11

**Authors:** Anup Barai, Kotub Uddin, Julie Chevalier, Gael H. Chouchelamane, Andrew McGordon, John Low, Paul Jennings

**Affiliations:** 1 WMG, University of Warwick, Coventry, CV4 7AL United Kingdom; 2 Hybrids and Electrification, Jaguar and Land Rover, Banbury Road, Warwick, CV35 0XJ United Kingdom

## Abstract

In freight classification, lithium-ion batteries are classed as dangerous goods and are therefore subject to stringent regulations and guidelines for certification for safe transport. One such guideline is the requirement for batteries to be at a state of charge of 30%. Under such conditions, a significant amount of the battery’s energy is stored; in the event of mismanagement, or indeed an airside incident, this energy can lead to ignition and a fire. In this work, we investigate the effect on the battery of removing 99.1% of the total stored energy. The performance of 8Ah C_6_/LiFePO_4_ pouch cells were measured following periods of calendar ageing at low voltages, at and well below the manufacturer’s recommended value. Battery degradation was monitored using impedance spectroscopy and capacity tests; the results show that the cells stored at 2.3 V exhibited no change in cell capacity after 90 days; resistance rise was negligible. Energy-dispersive X-ray spectroscopy results indicate that there was no significant copper dissolution. To test the safety of the batteries at low voltages, external short-circuit tests were performed on the cells. While the cells discharged to 2.3 V only exhibited a surface temperature rise of 6 °C, cells at higher voltages exhibited sparks, fumes and fire.

## Introduction

Lithium ion (Li-ion) batteries have become the electrochemical energy storage technology of choice in many applications due to their high specific energy density, high efficiency and long life. In tandem with rising demand for portable electronic devices as well as rapidly falling battery costs^1^, the global uptake of Li-ion batteries is increasing^2^. Carbon emissions legislation, in addition, is driving further significant demand for Li-ion batteries, which have gained prominence in renewable energy plants^3^, as well as energy storage systems for sustainable vehicles, such as hybrid and electric vehicles^4^. As the applicability of Li-ion batteries widens, market uptake increases. The demand for Li-ion batteries grew from circa. 49 GWh in 2013 to circa. 70 GWh in 2016 and is expected to rise to more than 96 GWh by 2020^5^.

The earliest, commercially available, rechargeable Li-ion batteries were based on cobalt cathodes i.e., LiCoO_2_ (LCO)^6^, Lithium Cobalt based batteries therefore dominated the rechargeable battery market in the last decade. Cycle life and safety concerns with this technology^7^ however, paved the way for batteries with Lithium Nickel Manganese Cobalt Oxide (NMC, LiNi_x_Mn_y_Co_z_O_2_) cathodes to dominate the market today. With safety concerns still associated with Cobalt^8, 9^ and the demand for even safer batteries, batteries based on lithium iron phosphate (LFP, LiFePO_4_) cathodes have gained significant prominence in the last few years. Lithium-ion Phosphate batteries (LiFePO_4_) are now employed in EVs such as the Fisker Karma range-extended electric vehicle, the GM spark EV and the BYD e6/s6DM.

Given that the production of lithium-ion batteries is heavily concentrated in South East Asia^10^, transportation of these devices to the majority of end users is a necessity. An industry-wide common practice is to adjust the battery’s state of charge (${SoC}$) to a value of 30% to 70% for safe transportation^11, 12^, which recently has recommended to limit to maximum 30% SoC by International Civil Aviation Organization (ICAO)^12^. Li-ion batteries are classified as dangerous goods and as such are required to pass section 38.3 of the UN Manual of Tests and Criteria (UN Transportation Testing) in order to be certified for transport^13^. The list of tests in UN 38.3 is shown in Table 1. Specific packaging instructions apply for road and sea transport, which are mainly categorised by the energy rating in Wh of the battery^13^. For a 100 Wh or smaller battery, SP188 applies. Under SP188, UN approved packaging is not a requirement for package sizes weighing less than 10 kg, provided a strong impact resistant case is used. For larger packages of up to 30 kg, the packaging must be made out of steel, aluminium, a metal other than steel or aluminium, rigid plastics, natural wood, plywood, reconstructed wood or rigid fibreboard, and withstand a 1.2 m drop test. For batteries larger than 100 Wh capacity, P903 applies and short circuit protection and UN approved packaging are among the necessary requirements for shipping. The battery needs to also be completely enclosed and there is a weight limit of 30 kg per package and 333 kg per vehicle^13^.

**Table 1:** UN 38.3 tests for transport certification of lithium-ion battery.

Test Number	Test Name	Short Description
UN 38.3.4.1	Test T.1	Cells and batteries stored at a pressure of 11.6 kPa or less for at least six hours at ambient temperature
	Altitude Simulation	
UN 38.3.4.2	Test T.2	Rapid thermal cycling between high (75 °C) and low (−40 °C) storage temperatures
	Thermal Cycling	
UN 38.3.4.3	Test T.3	Sinusoidal vibration pattern of 7 Hz with 1 g pack acceleration to 200 Hz with 8 g pack acceleration and back to 7 Hz; 12 cycles in three perpendicular mounting positions are applied
	Vibration	
UN 38.3.4.4	Test T.4	150 g shock for a duration of 6ms is applied in three different perpendicular positions
	Shock	
UN 38.3.4.5	Test T.5	Short circuit of less than 0.1 Ω at 55 °C for 1 hour is applied to the cell
	External Short-Circuit	
UN 38.3.4.6	Test T.6	15.8 mm diameter bar placed across cell center and a 9.1 kg mass is dropped onto the bar from 61 cm height
	Impact	
UN 38.3.4.7	Test T.7	More than double the recommended current and double the maximum voltage is used to charge the cell
	Overcharge	
UN 38.3.4.8	Test T.8	Over-discharge of the cell for a single instance
	Forced Discharge	

For air transportation of new batteries, which passed the UN 38.3 test, packaging guideline PI965 applies. For a 100 Wh or smaller battery, a weight limit of 10 kg per package applies and packaging needs to pass a 1.2 m drop test. For higher capacity batteries, the maximum net weight per package for cargo aircraft is 35 kg (PI965, SEC IA). However, there is a provision for large Li-ion batteries that have a net weight exceeding 35 kg; these need to be consigned on a cargo aircraft in accordance with air special provision A99. The consignment needs to be accompanied by documentation of approval by the appropriate authority in the state of origin.

Despite the regulations and provisions, there is a long history of air cargo transport incidents involving Li-ion batteries and devices employing Li-ion batteries. In Table 2, a comprehensive list of air cargo accidents attributed to Li-ion batteries is provided. This list comprises of incidents involving all Li-ion battery chemistries, including the most volatile LCO and relatively benign LFP batteries. This list excludes battery related air transport incidents associated with personal devices e.g. Samsung Galaxy Note 7 and batteries integrated into an aircraft e.g. the Boeing 787 Dreamliner battery fire issue.

**Table 2:** List of air-cargo/air-side transport incidents attributed to lithium-ion batteries and devices containing lithium-ion batteries^32, 41–45^.

Time	Incident	Root Cause
Oct-2011	Asiana airlines Cargo flight, a Boeing 747-400F, registration HL7604, crashed due to an in-flight fire in cargo bay.	Physical evidence did not permit identification of the exact cause of fire. However, the fire started near the pallets where lithium-ion batteries were being stored.
Sep-2010	UPS Airlines Flight 6, a Boeing 747-400F, registration N571UP, crashed due to an in-flight fire in cargo bay.	The fire was caused by the auto-ignition of the contents of a cargo pallet, which contained “a significant number” of lithium-ion batteries and other combustible materials
Aug-2009	FedEx discovered a burning and smoking package at one of their facilities, which contained GPS tracking devices with lithium-ion batteries, two of the devices had heated causing surrounding packaging and cushioning to ignite.	Mechanical shock/vibration, external short circuit improper packaging
Aug-2009	UPS found a smouldering package at its Taiwan Hub. Inspection of other packages in the same consignment indicated that similar batteries were shipped without terminal protection.	External short circuit, mechanical shock/vibration and improper packaging
July-2009	At the UPS hub in the Dominican Republic, a box started to emit smoke. The package had lithium-ion batteries for mobile phones.	External short-circuit of the lithium-ion batteries due to improper packaging
Aug-2008	UPS discovered a smoking package containing lithium-ion battery powered LED lamps at a ground sort facility.	External short circuit brought about by a combination of transport and handling shock and vibration with improper packaging
Dec-2007	Package containing a toy helicopter kit with lithium-ion polymer batteries was discovered emitting smoke at a FedEx sort facility.	External short circuit brought about by a combination of transport and handling shock and vibration with improper packaging
Sept-2007	At the Fedex facility package of lithium-ion battery was emitting smoke but the fire was contained within the box.	Mechanical damage
Jun-2007	A cargo hold fire alarm was activated during taxiing. The source was a package of lithium-ion battery.	External short-circuit
Aug-2004	A box containing lithium-ion battery module for prototype EV start to emit smoke on FedEx cargo plane loading ramp.	External short-circuit

Malaysia Airlines Flight 370 in 2014 was, later, confirmed to be carrying lithium-ion batteries in its cargo hold, sparking speculation that they may have caused a fire that brought the plane down. Similar suspicion, while not proven, arose for the EgyptAir Flight 804 that crashed in 2016. This points to ongoing concerns regarding the safety of transporting Li-ion batteries by air, therefore is still an open research question.

One way to make the transport of lithium-ion batteries safer is to remove the stored energy prior to transport. In this work, we investigate the viability of transporting Li-ion batteries, more specifically lithium iron phosphate (LFP) batteries, at voltages corresponding to 0% SoC and lower, i.e., after removing almost all of the energy stored in the electrochemical system. Irrespective of the lithium-ion cell chemistry, at extremely low cell voltages the potential of the graphite negative electrode (LiC_6_) increases significantly versus Li/Li^+^^14, 15^ and can lead to copper current collector dissolution^16–19^. Consequently, the dissolved copper ion can travel through the separator and be deposited, which leads to a growth of copper dendrite when cycled^19^. The copper dendrite can potentially create an internal short-circuit and compromise safety. Also, the corrosion of copper current collector creates a loss of mechanical and electrical contact between the current collector and the negative electrode components, leading to an increase in cell impedance^20^. The corrosion products, which have poor electronic conductivity, cause overpotentials; coupled with the loss of mechanical contact, this encourages inhomogeneous current (thus very high localized current) and potential distributions resulting in lithium dendrite growth^21^. The morphology of the cathode materials can also be changed at very low potentials, below 1 V. The side reactions that occur during extreme overdischarge result in the solid-state amorphization of the transition metal compounds^17^. The changes in electrode morphology leads to capacity degradation^19^. Under extremely low voltages, these electrochemical processes are present in LFP based cells as well as other lithium-ion battery chemistries alike.

In this paper, after studying the effects of long term, low voltage storage on the performance of LFP cells, the safety of LFP cells at such low voltages when exposed to external short-circuit conditions (the most common cause of the incidents in Table 2) is studied. It is shown that a voltage stability window exists where the degradation associated with storing the battery at low voltages is negligible; concurrently the battery is effectively “inert” because the energy stored in the electrochemical system is almost entirely extracted. Transporting batteries under such conditions would be relatively safer than the adopted industry practice today, with little cost in terms of degrading long-term battery functionality. The relatively benign LFP cells were chosen for this pilot study because even this benign chemistry may have the potential to create a fire during an external short circuit. Since the ageing mechanisms at low SoC are common between all Li-ion battery chemistries and the potential for a fire hazard is related to the stored energy, the high level conclusions derived from studying LFP cells may extrapolate to other Li-ion cell chemistries. A detailed investigation of other Li-ion cell chemistries in this regard will be addressed in future studies. The experimental procedure adopted for this study is presented in the ‘experimental details’ section. The ‘Low voltage calendar ageing results’ section presents the long-term low voltage storage ageing results. The conclusions from this section were used to identify the optimum ageing condition to take forward to the external short-circuit test described in ‘short circuit abuse experiment’ section. An overall discussion on ageing test results and short-circuit test results are presented before summarizing the key contributions.

## Experimental details

Commercially available Li-ion pouch cells with a LiFePO_4_ (LFP) cathode and LiC_6_ (graphite) anode were used for this study. The rated capacity and maximum discharge current limits of the cells were 8 Ah and 40 A, respectively and weight 0.157 kg. The maximum cell voltage during charging is specified by the manufacturer to be 3.65 V, while it is 3.4 V under the constant-current—constant-voltage (CC-CV) charging protocol; similarly, the minimum discharge cut-off voltage is 2.3 V. To isolate the effect of temperature, all the tests were carried out at 25 °C within a temperature controlled environmental chamber.

To capture the electrical performance of the cell, a set of characterisation tests (snapshot tests) were performed on the cells at the beginning of the test. The snapshot test comprised of 1 C capacity charge and discharge tests and electrical impedance spectroscopy (EIS) tests. At the beginning of the discharge capacity test, the cells were discharged at a 1 C rate to 2.3 V using a Bitrode MCV 16-100-5 Li-ion cell cycler. The cells were then allowed to rest for 2 hours before being fully recharged via the CC-CV protocol using a C/3 current for the CC part, to 3.4 V and a C/20 cut-off rate for the CV part. Following a further 2 hours of rest, the cells were discharged using the 1 C current rate. EIS tests were performed at 50% SoC using a Solartron Modulab system (model 2100 A) fitted with a 2 A booster card. Impedance measurements between 10 mHz and 10 kHz with 10 frequency points per decade were taken. The applied amplitude (RMS value) of the signal was 800 mA. A minimum of 4 hr rest was allowed after SoC adjustment before performing EIS measurements^22^.

Following an initial snapshot test, the cells were discharged to 2.3, 2.0, 1.0 and 0.5 V and then held at that voltage for 15 days. After 15 days of storage at constant voltage, another snapshot test was performed and the constant voltage storage continued. These four different storage voltages were chosen to identify the optimal voltage, which minimises battery degradation. Three cells per storage condition were used to ensure statistical significance and to reduce the impact of cell-to-cell variations, thus a total of 12 cells were used for this experiment.

### Low voltage calendar ageing results

**Figure 1:**
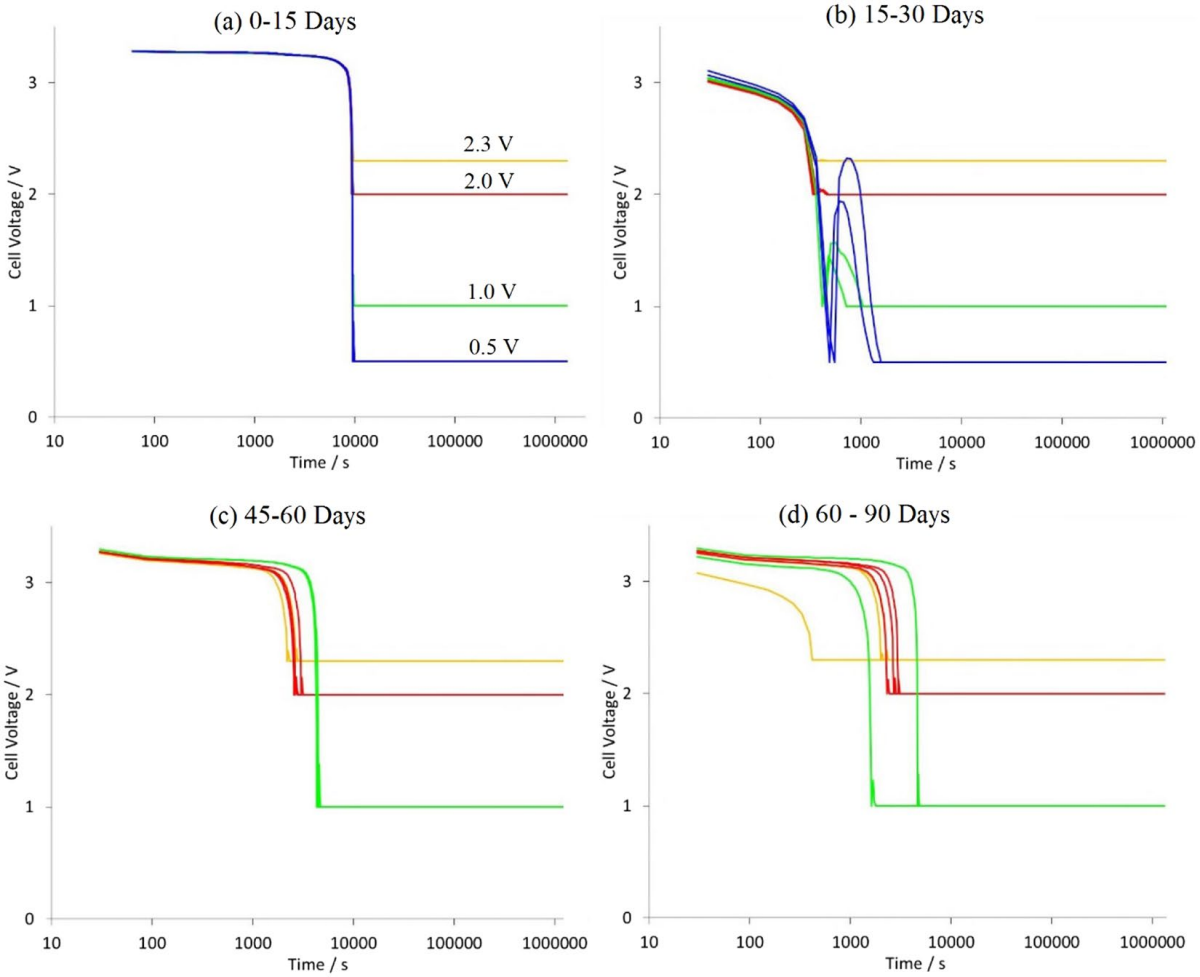
Discharge voltage and constant voltage storage for all cells. Cells stored at 0.5 V became dysfunctional after 30 days.

Figure 1 shows cell voltage as the cells were discharged to 2.3, 2.0, 1.0 and 0.5 V using 1 C current following each snapshot test. The energy extracted by discharging to a lower voltage compared to the manufacturer recommended 2.3 V, is shown in Table 3. The extra energy extracted by discharging from 2.3 V to 2 V amounted to only 0.3% of the total energy extracted, while discharging to 0.5 V yielded an extra 0.9% of the total energy. Note that while SoE varied by 0.9%, the corresponding SoC variation was 1.9%, which is due to the fact that SoC is calculated from coulomb counting, whereas SoE is additionally governed by the cells voltage. Thus, due to the falling voltage with discharge, the cell delivers progressively lower amounts of energy (SoE) for every unit of charge (SoC), further details can be found in the work of *Barai et al*.^23^.

**Table 3:** Showing the energy extracted in Wh and Ah by discharging to each voltage in comparison to discharging to the manufacturer recommended 2.3 V.

Cell Voltage	Cell 1		Cell 2		Cell 3		Average		SoE	SoC
	Wh	Ah	Wh	Ah	Wh	Ah	Wh	Ah		
2.3	24.62	7.77	24.66	7.77	24.89	7.85	24.72	7.80	**0.0%**	**0.0%**
2.0	24.70	7.80	24.74	7.81	24.95	7.87	24.80	7.83	**−0.3%**	**−0.4%**
1.0	24.80	7.88	24.86	7.89	25.07	7.96	24.91	7.91	**−0.8%**	**−1.5%**
0.5	24.84	7.91	24.89	7.92	25.10	7.99	24.94	7.94	**−0.9%**	**−1.9%**

The difference is calculated using the average of three cells.

In Fig. 1(b) the voltage is seen to momentarily relax to a higher value just after the cells were discharged to 0.5 V and 1.0 V. This anomaly was due to the experimental setup and as was corrected within 7 minutes. This period of elevated voltage exposure has negligible effect since a few minutes at marginally elevated voltages, relative to the storage duration of 15 days, constitutes less than 0.01% testing time.

**Figure 2:**
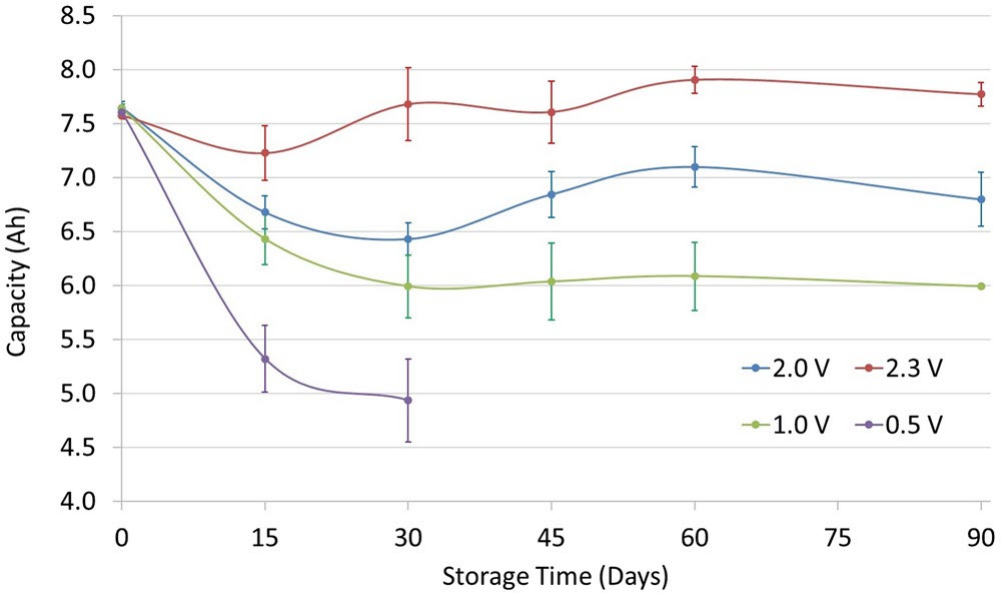
Capacity fade with storage duration at different voltage.

The capacity of the cells from each snapshot test is shown in Fig. 2. Due to significant degradation observed for the 0.5 V scenario at the 3^rd^ snapshot test (after 30 days of storage), this test was discontinued. After 15 days of storage there was a sharp capacity drop, which was higher for lower storage voltages. Capacity dropped by more than 35% after 30 days of storage at 0.5 V, which posed a safety risk (explained later in this section) and therefore the test was not continued. After 15 days, capacity drop was 30.1%, 15.8%, 12.6% and 4.6% for 0.5 V, 1.0 V, 2.0 V and 2.3 V test conditions respectively. Capacity continued to drop for cells stored at 1.0 V and 2.0 V up to 30 days of storage. After this point, capacity fade stabilised for cells stored at 1.0 V. In contrast, the 2.0 V storage results exhibited a marginal increase in storage capacity post 30 days, rising to a capacity fade of 7.1%. After 90 days of storage at 2.0 V the final capacity fade was 11.1%. Cells stored at 2.3 V exhibited capacity increase after 15 days, although within the error bar (due to cell to cell variation). However, there was a confirmed overall rise of 2.6% in cell capacity after 90 days for all three cells stored at 2.3 V. This is consistent with previous studies such as that that of Kassem *et al*.^24^ and Li *et al*.^25^. In the latter study, the authors stored C_6_/LiFePO_4_ batteries at room temperature; for cells stored at ≤20% SoC, they reported an increase in cell capacity. In summary, drawing on calendar life studies reported in refs 24–27, storing at 2.3 V for 90 days leads to less capacity fade than storing at ~30% SoC, which is the current aviation standard.

**Figure 3:**
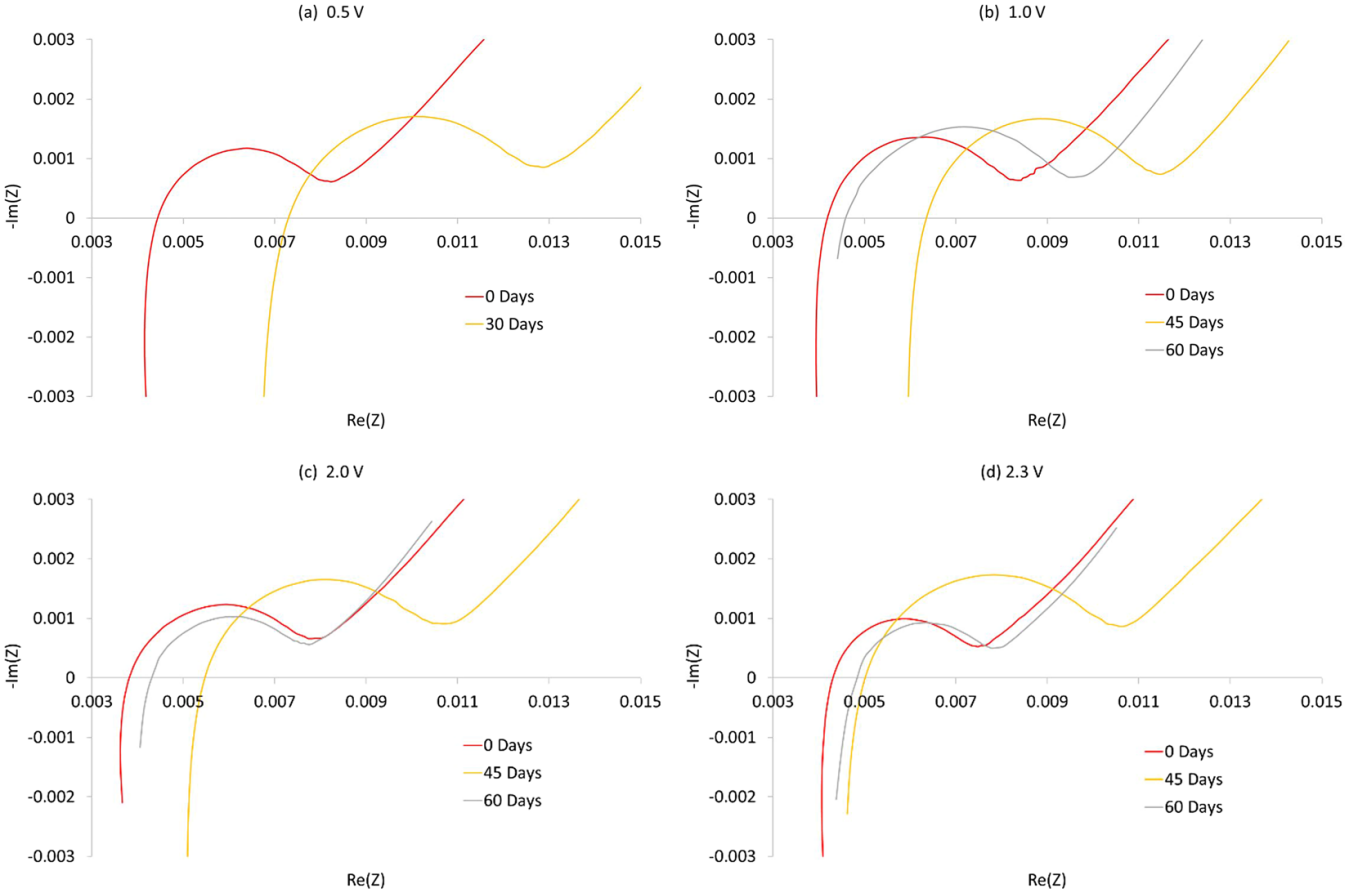
Change of impedance measured by EIS at 50% SoC. The x-axis has the real part of the complex impedance and y-axis has the imaginary part, both has unit of Ω.

The EIS test results at 50% SoC in the form of Nyquist plots are shown in Fig. 3. The x-axis has the real part of the complex impedance and y-axis has the imaginary part of the complex impedance, both has unit of Ω. In agreement with the capacity results, the pure Ohmic resistance, ${R}_{o}$, first increases and then stabilises with a slight decrease. After 30 days of storage there is an increase in Ohmic resistance for every storage condition; the 0.5 V storage condition exhibited the highest increase (3 mΩ); the lowest increase (1 mΩ) was found for the cell stored at 2.3 V. After 45 days of storage there was a clear rise in total resistance ${R}_{t}$, as shown in Fig. 3(b),(c) and (d). After 60 days the ${R}_{t}$ value for storage at 2.3 V dropped close to its initial value.

The Ohmic resistance (${R}_{o}$) includes both ionic resistance of the electrolyte and electronic resistance of the electrode and current collectors^28–31^. Under low voltage storage, it is reported that the copper current collector reacts with electrolyte components resulting in corrosion^16–18^, leading to higher ${R}_{o}$. More specifically, when cells are stored at low voltages over an extended period, the copper current collector attached to the carbon electrode is oxidised to Cu^2+^ and dissolves into the electrolyte^14, 19, 32^. The subsequent reduction in contact between the current collector and active electrode material manifests as an increase in Ohmic resistance. Although previous studies such as that of Jeevarajan *et al*.^33^ reported slight increases of cell resistance under over-discharge conditions, the onset and rate of copper dissolution is cell specific.

To assess and quantify copper dissolution under low voltage storage for the cells studied in this work, energy-dispersive X-ray spectroscopy (EDX) was used. Samples of negative electrode material from aged cells were extracted in a glove-box, within an argon environment. For robustness, two samples were taken from each cell. The elemental composition of the electrode samples was analysed; the EDX spectrum showed very small traces (~1%) of copper in all cases. Given that the EDX technique has a widely accepted tolerance of ~5%, no conclusive observation can be made from these results.

Under over-discharge conditions, over-deintercalation of lithium at the negative electrode can cause decomposition of the solid electrolyte interphase (SEI). When the cell is re-charged, new SEI film forms on the graphite anode. The growth of the SEI film leads to degradation of the electrochemical charge-transfer processes at the electrode-electrolyte interface^15, 34, 35^. Furthermore, the decomposition of SEI leads to gas generation at the negative electrode. The generation of gases, typically CO_2_ and CO, cause swelling within the cell^14^ and consequently a resistance rise due to electrical contact loss thorough delamination. Cell delamination varies with the volume of gas generated and post experimental cell relaxation time. In order to perform the EIS tests presented in Fig. 3, cell SoC was adjusted to 50% SoC. The magnitude of cell resistance reduction due to the decrease in cell volume expansion depends on how long the cell spent at 50% SoC. This explains the falling resistance observed in Fig. 3.

At the cathode, when the cell is discharged to below the voltage limit, irreversible breakdown of LiFePO_4_ active material also occurs, which also releases gas^24^. This breakdown of active material leads to capacity fade. Kassem *et al*.^24^ reported a reversible capacity loss due to side reactions at the positive electrode, which was further explained by Li *et al*.^25^. The reversible and irreversible capacity fade found at different storage voltages and durations may be associated with these two mechanisms, SEI formation and gas evolution.

Results of the long term ageing results presented here, namely that copper dissolution is negligible and the principal mode of degradation is electrolyte decomposition and subsequent SEI growth, is consistent with the results reported by Guo *et al*.^19^. Guo *et al*. found that the dissolution of SEI occurs within 0 to −10% SoC, severe copper dissolution then occurs below −12% SoC, with severe internal short circuiting occurring at or lower −20% SoC^19^. For the batteries used in this study, 0.5 V corresponds to −1.9% SoC and as such, in agreement with Guo *et al*.^19^, only SEI dissolution and gassing occurs.

The battery degradation results presented in this section suggests a voltage stability window between 2.0 V and 2.3 V (−0.4% to 0% SoC) where the discharge voltage leads to a minimal effect on cell ageing. However, practical abuse testing is still required to investigate whether this voltage represent an improvement in safety. The 2.3 V condition was chosen for abuse testing as it has a higher remaining energy than other conditions where cells were discharged to a lower voltage. If the cell is shown to be inert at 2.3 V, then this inertness will hold at lower voltages.

### Short Circuit Abuse Experiment

A lithium-ion cell which is discharged to −1.9% ≤SoC ≤0% (as done in this study) is expected to be safer to transport than at higher SoC conditions. While the toxicity of the active material within the cell remains the same, it is less likely to self-ignite due to internal/external short-circuits or even under a crash scenario. Within this SoC window the batteries have less stored energy; under a failure scenario, the cells are likely to produce less heat and thus the probability of reaching thermal runway is significantly lower^36–38^. If a cell reaches thermal runway, the stored chemical energy will be released, which may lead to an explosion^9^.

**Figure 4:**
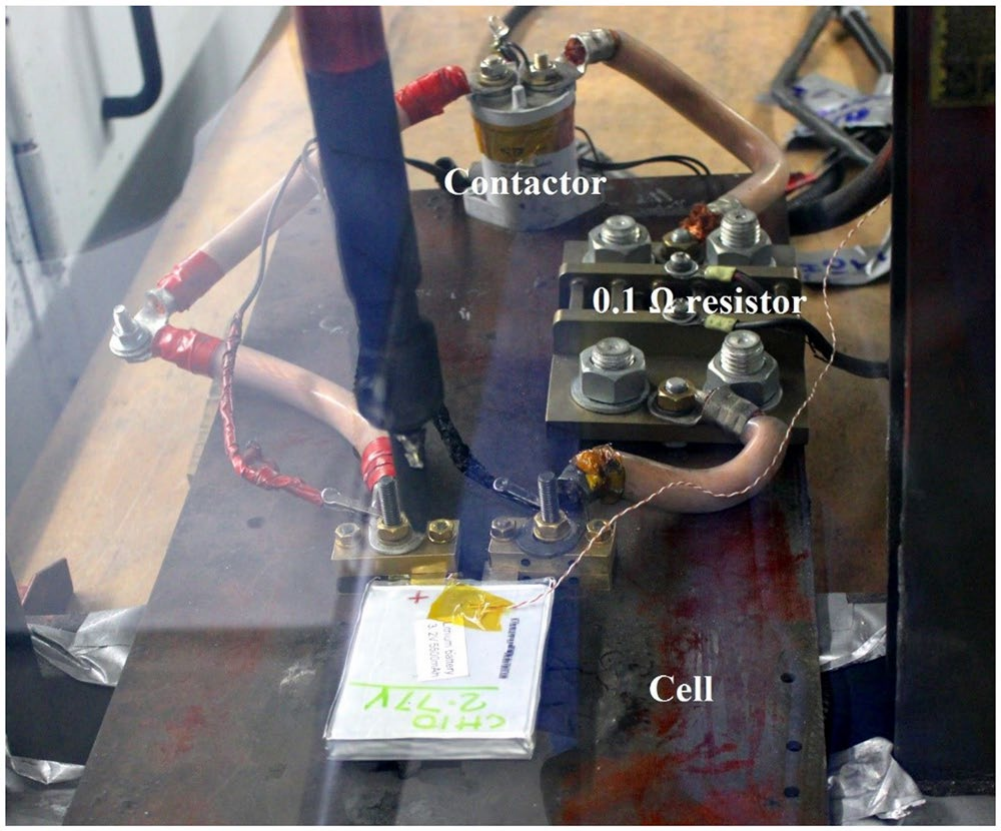
External short circuit test setup.

To validate the low voltage transportation protocol proposed in this paper, external short-circuit tests were performed at different SoCs. An external short-circuit is one of the most common reasons (Table 2) for lithium-ion battery failure/incidents during transport and therefore it was chosen to mimic a real failure condition. For this test, a new set of 15 cells were used. The first batch of three cells were stored as supplied with around 60% SoC; SoC of subsequent batches of three cells were adjusted to 70%, 30% and 5% SoC; the 5^th^ batch of three cells were discharged to 2.3 V (0% SoC). The test setup is shown in Fig. 4. An external short circuit was applied to the cell using thick copper cables and a contactor synchronised with a data acquisition system; contactor was used to close the circuit remotely. A 0.1 Ω resistor was embedded into the current path to measure current. The test was completed within a purpose-built chamber for abuse testing of high energy storage systems. The test was performed at room temperature (25 ± 3 °C). Video recording and cell surface temperature measurements were made during the test.

**Figure 5:**
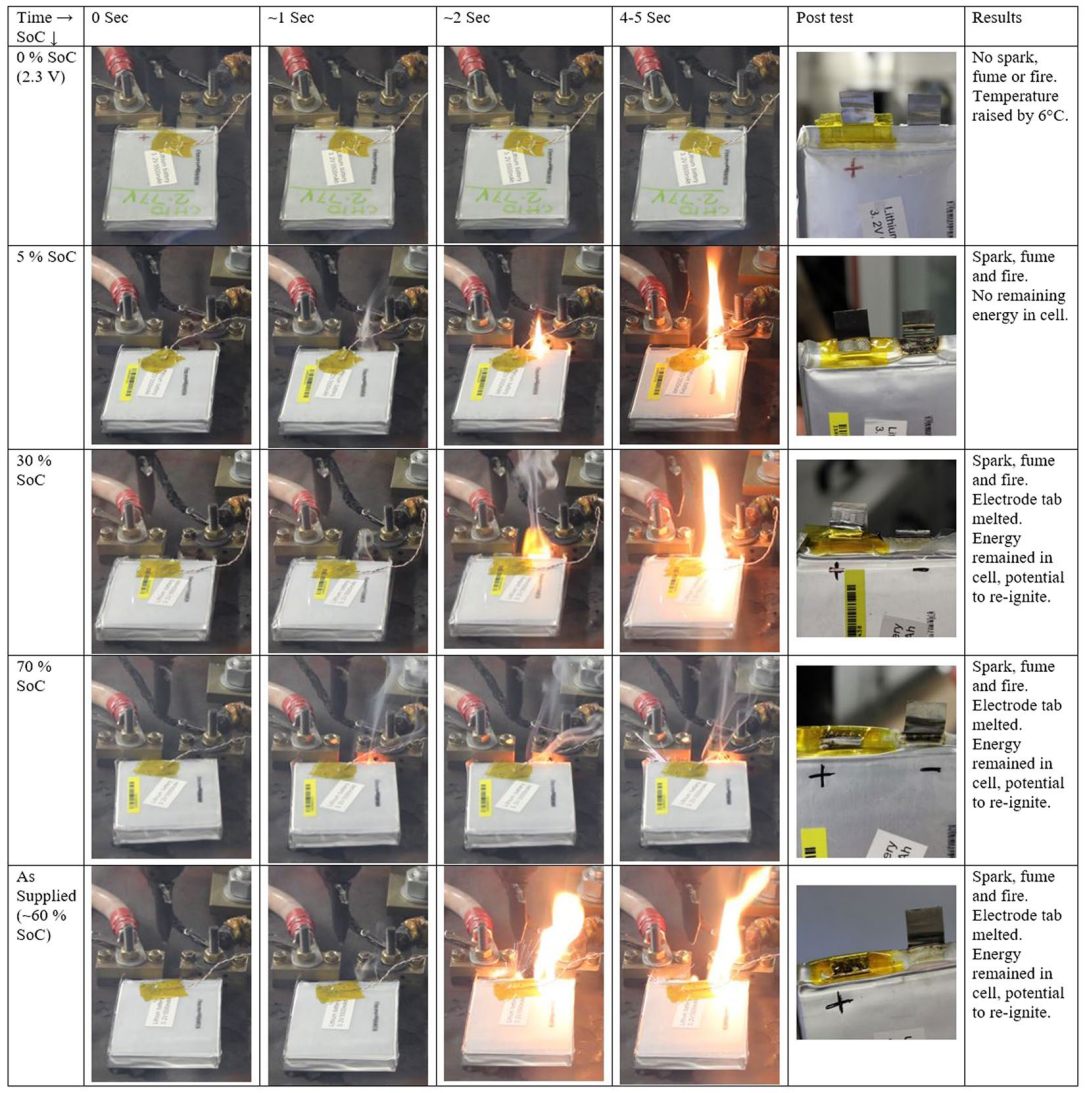
External short-circuit test performed on five cells at 0% SoC (discharged to 2.3 V), 5% SoC, 30% SoC, 70% SoC and as supplied (around 60% SoC) by the manufacturer. From left to right, first picture is just before the short-circuit was applied, just after application of short circuit (~1 sec), 2 second after application of short circuit, maximum fire/spark found (around 4–5 seconds after the short circuit was applied) and in last picture the cell electrode tab after short-circuit test. Results of the test are presented in last column.

Snapshots of the cell taken during the short-circuit tests are presented in Fig. 5. Video recordings capturing the cell response to a short-circuit can be found in the online version of the paper under supplementary information. The cell which was discharged to 2.3 V did not have any sparks, fumes or fire (Fig. 5). The temperature rose by 6 °C. In contrast, cells with SoC ranging from 5% to 70% exhibited sparks and the cell enclosure near to the electrode tab caught fire. In addition, the tabs of the cells with the highest SoC melted and fused under a short-circuit, see Fig. 5. These cells eventually could not discharge, which meant that considerable energy was still stored within the cell and posed a risk of further short-circuiting. Although the cells with 5% SoC had enough energy stored to create a fire, it was not enough to fuse the electrode tab material; however, clearly any sort of fire is undesirable on an aircraft.

It is important to note that the fire which ignited the cell’s outer packaging close to the cells electrode tab did not cause an ignition of the cell’s active material in any of the cases considered in this work. Moreover, cell temperatures did not reach a high enough level to ignite the active material within the cell. Although it could be argued that an effective discharge under extremely high currents could have led to an internal short circuit due to lithium plating^21^, there were no external signs (such as swelling) of an internal short-circuit.

## Discussion

As mentioned earlier, battery SoC is adjusted to a value between 30% and 70% at the end of cell production. This is mainly due to the speculation within the battery industry that calendar ageing of Li-ion cells are accelerated when stored at low SoC. However, the authors have found little evidence in literature to support this claim. In contrast, there is evidence that battery ageing is minimised when stored at low SoC^25^. If these batteries need to be shipped via sea, then they are subject to calendar aging. A regular shipment from China to UK typically takes about 6–8 weeks. On the voyage, the temperature within a regular cargo will vary depending on the route and time of the year. Based on calendar life studies of lithium-ion cells^24, 26, 39, 40^, an ageing of 2–5% (depending on temperature) is expected for this duration when stored between 30% and 70% SoC. In contrast air-freight takes only days, but currently is considered dangerous cargo and have led to several incidents. In a viable transportation scenario, safety is maximised with no compromise to the functionality of the system. This work presents results showing that cells which are discharged to 0% SoC or lower become inert and cannot create a fire even under a short-circuit scenario. Such conditions are therefore conducive to safer transportation of Li-ion batteries. Although storing batteries at SoCs below 0% SoC, i.e., at significantly low voltages, is ideal from a transport safety point of view, it was found that SEI dissolution and gassing persevere under such conditions causing irreversible ageing. At 0% SoC however, cells only exhibited reversible capacity loss, and therefore the adverse effects on functionality due to long term ageing can be avoided. Although long-term calendar life studies at 0% SoC (and lower), such as the work presented in this paper, is not reported in literature, Li *et al*.^25^ reported calendar ageing at 10% SoC which shows similar trends as found here at 0% SoC, namely that there is an apparent rise in capacity. Furthermore, the results reported here are in-line with the electrochemical mechanisms of degradation for extremely low SoCs ($\le$0%) described by Guo *et al*.^19^. Therefore, a voltage stability window around 0% SoC exist, where the cell degradation is minimal. In summary, discharging Li-ion cells to 0% SoC, can be adopted as a standard for transpiration of lithium ion batteries. Even cells discharged to 5% SoC exhibited fire, highlighting that cells need to be discharged to 0% SoC, well below the 30% SoC standard. If safe air freight is possible, it will accelerate the development and production of EV battery packs, and reduce transportation costs.

These conclusions based on the LFP cells may persist for other cell chemistries as well, as firstly, the fire created in an external short-circuit event is due to the stored energy and if the energy is removed from any cell they will simply become inert. Secondly, when cells are stored after removing energy, the ageing is dominated by the negative electrode, which in most commercial batteries is graphite, LiC_6_, hence other Li-ion battery chemistry cells will also likely not age at such low SoC conditions – although this point requires further investigation. Hence, a number of opportunities exist where the research presented here may be further extended and refined. Although it is estimated that the conclusions will persist for other Li-ion cell chemistries, validation with various cell chemistries and form factors is required to establish conclusive proof. Also, given the limited datasets employed for this initial study, further experiments within the 0% to 5% SoC window will precisely identify the best SoC point to store the cells for transport. A detailed electrochemical study into gassing and resulting degradation on the cells stored at low SoC will be a natural extension of this work. This will investigate if there is any phase change occurring at low voltage^16^.

It should be noted that although by discharging the cells to 0% SoC, the stored electrical/electrochemical energy is predominantly removed, chemical energy stored within the bonds of the chemical compounds of component martials still exist. As such, under abuse conditions which will stimulate extensive exothermic chemical reactions, such as aggressive thermal insult, the hazard of thermal runway and combustion^9^ still exists.

## Conclusion

Considering the challenges facing long-haul transportation of Li-ion batteries, in this paper we propose a protocol whereby 99.1% of the battery’s energy is removed prior to shipping. We show that removing 99.1% of the total stored energy (0% SoC) of a Li-ion battery of LFP chemistry is safer than the current ICAO standard of 30% SoC for transportation in the event of short circuit. Using a novel dataset, it was shown that cells stored at such low SoC values did not exhibit significant irreversible capacity fade. While storing at very low voltages ($\le$0.5 V per cell) is ideal from an electrical hazard perspective, the results indicate, in agreement with previous literature, that SEI dissolution was more pronounced, leading to significant degradation of battery capacity (up-to 30% within 15 days). On the other hand, around 0% SoC was found to be a voltage stability window for the transportation of Li-ion batteries, which does not comprise the battery’s state of health.

An external short-circuit test was performed on the cells to validate the proposed safer transport protocol where the SoC is discharged to 0% SoC. The external short-circuit tests on the cells at different SoC from 5% to 70% exhibited sparks, fuming and even fire. However, the cells discharged to 2.3 V (0% SoC) did not show any of these signs; only exhibiting a surface temperature rise of 6 °C. The paper presented discussion on why these conclusions may still be valid for other Li-ion battery chemistries.

This research provides evidence that safer air-freight is possible by removing almost all of a cell’s stored energy. While there are other measures such as stringent packaging standard, that can be employed to reduce the hazard, the method proposed in this work efficiently removes the hazard, enabling safer transport of Li-ion batteries.
